# Dynamic contrast enhanced magnetic resonance imaging in chronic Achilles tendinosis

**DOI:** 10.1186/1471-2342-13-39

**Published:** 2013-11-22

**Authors:** Anna Gärdin, Torkel B Brismar, Tomas Movin, Adel Shalabi

**Affiliations:** 1 Department of Clinical Science, Division of Medical Imaging and Technology, Intervention and Technology at Karolinska Institutet, Stockholm SE-141-86, Sweden; 2 Department of Radiology, Karolinska University Hospital in Huddinge, Stockholm SE-141-86, Sweden; 3 Department of Clinical Science, Division of Orthopedics and Biotechnology, Intervention and Technology at Karolinska Institutet, Stockholm SE-141-86, Sweden; 4 Centre for Medical Imaging, Department of Radiology, Oncology and Radiation Sciences Akademiska University Hospital, Uppsala SE-751-85, Sweden

## Abstract

**Background:**

Chronic Achilles tendinosis is a common problem. When evaluating and comparing different therapies there is a need for reliable imaging methods. Our aim was to evaluate if chronic Achilles tendinosis affects the dynamic contrast-enhancement in the tendon and its surroundings and if short-term eccentric calf-muscle training normalizes the dynamic contrast-enhancement.

**Methods:**

20 patients with chronic Achilles tendinopathy were included. Median duration of symptoms was 31 months (range 6 to 120 months). Both Achilles tendons were examined with dynamic contrast enhanced MRI before and after a 12- week exercise programme of eccentric calf-muscle training. The dynamic MRI was evaluated in tendon, vessel and in fat ventrally of tendon. Area under the curve (AUC), time to peak of signal, signal increase per second (SI/s) and increase in signal between start and peak as a percentage (SI%) was calculated. Pain and performance were evaluated using a questionnaire.

**Results:**

In the fat ventrally of the tendon, dynamic contrast enhancement was significantly higher in the symptomatic leg compared to the contralateral non-symptomatic leg before but not after treatment. Despite decreased pain and improved performance there was no significant change of dynamic contrast enhancement in symptomatic tendons after treatment.

**Conclusion:**

In Achilles tendinosis there is an increased contrast enhancement in the fat ventrally of the tendon. The lack of correlation with symptoms and the lack of significant changes in tendon contrast enhancement parameters do however indicate that dynamic enhanced MRI is currently not a useful method to evaluate chronic Achilles tendinosis.

## Background

Chronic Achilles tendinosis is common especially in athletes but also in non- athletes [1,2]. Deranged vasculature is thought to be involved in the pathogenesis [3]. A pathologic vascularity not only inside the tendon but also outside, ventrally of the tendon, has been shown [4]. Several treatment approaches have been applied [5-9]. When evaluating and comparing different therapies there is a need of reliable imaging methods since evaluation with clinical examination and questionnaires cannot be fully blinded. By using ultrasound tendon thickening, hypoechoic areas and neovascularisation can be visualized and quantified [4,10,11]. Unfortunately ultrasound is user dependent and reproducibility is limited [12]. MRI techniques focus on measuring tendon thickening and on quantifying areas of intratendinous high signal [10,11,13]. By also administering intravenous gadolinium contrast media the intratendinous signal changes have been shown to be more pronounced [11]. After histopathological correlates it was hypothesized that the increased enhancement of the affected tendons from intravenous gadolinium might be explained by a local pathologically increased amount of glycosaminoglycans, a macromolecule with high water containing capacity.

In a previous publication we have shown that the absolute signal intensity was increased in symptomatic tendons before training and that it decreases towards normal values after three months of eccentric training [14]. Comparison of intratendinous signal between different occasions is however not fully reliable due to its arbitrary scaling with different extrinsic factors, such as placement of coils, temperature and normal T1/T2 fluctuations affecting the signal intensity. To avoid that methodological error a semi-quantitative scale was used when performing a follow-up of these patients 4 years after training [15]. The intratendionous signal of the previously affected tendons was then comparable to that of the non-symptomatic tendons. Use of semi-quantitative scales does however have a lower sensitivity than quantitative measurements due to influence from inter- and intra-observer variation. Further improvements of techniques to quantify Achilles tendinosis are therefore needed.

At dynamic contrast-enhanced MR imaging (DEMRI) of the Achilles tendon several images are obtained at fixed time-points after the injection of an MRI contrast agent. By analyzing the changes in signal intensity it is possible to calculate the physiologic kinetics. This gives information about tissue vascularity, perfusion and capillary permeability [16,17]. This technique should in theory be ideal to quantify the histopathological changes that have been observed in chronic Achilles tendinosis including increased vascularity, abnormal fiber structure, focal variation in cellularity and increased areas of non-collagen extracellular matrix [18,19]. The micro vascularisation in Achilles tendinosis has been evaluated during one year in a pilot study using MRI time-intensity curves together with power Doppler ultrasound [20].

The aim of this study was to evaluate the dynamic contrast-enhancement in Achilles tendinosis and if an altered dynamic contrast-enhancement is normalized after a short-term eccentric calf-muscle training. We also evaluated if the dynamic contrast-enhancement is correlated to the subjective symptoms.

### Patients

This study consists of a subgroup of a previously published patient material [14]. In that study intratendinous MRI signal, tendon volume and pain and performance before and after three months of eccentric training was evaluated. That study and the present study were approved by the local ethics committee at Karolinska Institutet and informed consent was obtained from each patient.

Because of technical problems when performing the DEMRI (three cases of incorrect contrast injection timing, one with extravasation and in one case intolerance of the contrast agent) only 20 patients of the original 25 patients participated in this dynamic contrast study.

The 20 patients (14 male and 6 females) were aged 28 to 70 years (median 51). Achilles tendinosis was defined as pain and local tenderness of the midportion of the Achilles tendon 2 to 7 cm proximal to the tendon insertion. Median duration of symptoms was 31 months (range 6 to 120 months). In 9 of the 20 patients the Achilles tendon symptoms were sports related; at the time when symptoms appeared 7 patients practiced recreational running and one of them also practiced skiing and another also basketball. One patient practiced golf and one patient related the symptoms to snowboard skiing. The remaining 11 were regarded as idiopathic. Previous corticosteroid injections and systemic diseases such as rheumatic disease constituted exclusion criteria. 14 of the 20 patients had unilateral symptoms whereas 6 of the patients also had a history of symptoms in the contralateral Achilles tendon. In patients with bilateral symptoms, the Achilles tendon with most symptoms was considered as the symptomatic tendon. One of the 20 patients had had an operation in the contralateral tendon 5 years previous to this study. None of the other patients had had any previous treatment other than pain medication.

### Treatment

The eccentric exercise programme followed a model developed by Alfredson et al. [5]. Eccentric loading of the calf was done in two ways; with the knee straight and with the knee bent. The eccentric loading was performed with all body weight on the symptomatic side. Each of the two exercises was done with 15 repetitions in 3 sets (3 × 15 repetitions) twice a day for 12 weeks. The patients were instructed to train their symptomatic side, but it is possible that some patients might have also trained the non-symptomatic side. To ensure compliance the patients saw a physiotherapist on two occasions and were contacted once on telephone. Symptoms were evaluated using a questionnaire, modified from a classification by Curwin and Stanish, grading pain on a six-level scale and performance on a four-level scale [21] (Table 1).

**Table 1:** Classification of the level of pain and functional impairment

**Intensity**	**Level**	**Pain**	**Level**	**Performance**
**Mild**	1	None	1	Normal
	2	With extreme exertion only, not intense	1	Normal
**Moderate**	3	Starts with activity, lasts for 1–2 hours after activity	2	Performance may be affected
	4	With any athletic activity, increase during activity	3	Performance level significantly decreased
**Severe**	5	Immediately upon any activity involving tendon. Sudden increase in pain if activity is continued, lasts for 12–24 hours	3	Performance markedly curtailed or prevented
	6	During daily activities	4	Unable to participate

Modified from Curwin & Stanish [18].

### MRI acquisition

Both Achilles tendons were examined with Dynamic MRI at two occasions, before (DEMRI 1) and after three months of heavy loaded eccentric calf-muscle training (DEMRI 2) using a 1.5 Tesla Magnetom Vision (Siemens, Erlangen, Germany). Both Achilles tendons were examined simultaneously using a commercially available CP-flexible 21 × 52 cm coil centered over the Achilles tendons. The patients were examined in a supine position with the feet in a resting position in the coil. The sequence used for the dynamic MRI series was a sagittal Flash 2D with 3 mm slice thickness, TR/TE 50/9 ms, flip angle 40°, FOV 180 × 180 mm, matrix 128 × 256 mm, time of acquisition 31 seconds. The contrast agent (gadopentetate dimeglumine, Magnevist®, Schering or gadodiamid, Omniscan®, Nycomed Amersham), 0.2 mmol/kg body weight, was injected by an auto-injector, 2 ml/second, via a catheter in the cubital vein followed by 20 ml of saline. The acquisition of the Dynamic MR series was started at the same time as the contrast injection. The Dynamic MR series lasted 5 minutes and 20 seconds, consisting of 5 sets with an interval of 40-seconds followed by two sets with an interval of 80 seconds. Every set had four sagittal slices, two from the symptomatic tendon and two from the contralateral tendon.

### Evaluation of dynamic MRI

**Figure 1:**
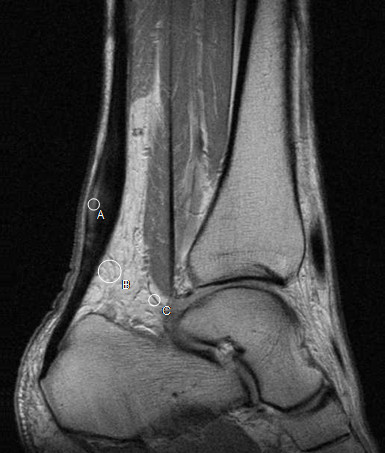
**Proton density weighted turbo spin-echo image of the right Achilles tendon prior to eccentric training in a 45-year-old man with chronic Achilles tendinosis.** There is a fusiform thickening of the mid-portion of the Achilles tendon with increased intratendinous signal. The measurement regions of interest were placed in: **A** = the tendon, **B** = the fat ventrally of the tendon and **C** = a vessel.

The Dynamic MRI series of the symptomatic and the contralateral tendons, before and after three months of eccentric training, were evaluated on a Hermes workstation (Nuclear Diagnostics, Stockholm, Sweden). From the two sagittal slices obtained from each Achilles tendon in every set, the most centered one was chosen by visual estimation. A circular ROI with a radius of 2 mm was placed in the thickest part of the tendon, and in the horizontal direction the area with the highest signal was chosen (Figure 1). In the case of no visible signal increase the ROI was placed in the centre of the thickest part of the tendon. The ROI’s distance from the upper posterior margin of the calcaneal bone was noted to ensure correct placement of the ROI at the follow up examination. One ROI with a radius of 2 mm was placed in a vessel ventrally of the tendon. One ROI with a radius of 5 mm was placed in the fat ventrally of the tendon. The three ROI:s placed in the first image of the dynamic series were then copied and pasted into the following images of the dynamic series. The signal intensity of each ROI was then plotted as a function of time, reflecting the contrast enhancement. Since signal intensity is a relative value and changes proportionally, the signal intensity at each point of the dynamic series was normalized by dividing the signal intensity of all subsequent intensity values with that obtained at the start of contrast injection (SI_0_) (i.e. so that SI_0_ = 1). The area under the curve (AUC) was then calculated for each ROI from the start of contrast injection until 320 seconds later, using the trapezoid method [22].

$$\begin{array}{l} \mathrm{AUC}=\left({\mathrm{SI}}_{0}+{\mathrm{SI}}_{1}\right)\times 40\;\mathrm{seconds}/2\\ \;+\left({\mathrm{SI}}_{1}+{\mathrm{SI}}_{2}\right)\times 40\;\mathrm{seconds}/2\\ \;+\left({\mathrm{SI}}_{2}+{\mathrm{SI}}_{3}\right)\times 40\;\mathrm{seconds}/2\\ \;+\left({\mathrm{SI}}_{3}+{\mathrm{SI}}_{4}\right)\times 40\;\mathrm{seconds}/2\\ \;+\left({\mathrm{SI}}_{4}+{\mathrm{SI}}_{5}\right)\times 80\;\mathrm{seconds}/2\\ \;+\left({\mathrm{SI}}_{5}+{\mathrm{SI}}_{6}\right)\times 80\;\mathrm{seconds}/2\\ \;-{\mathrm{SI}}_{0}\times 320\;\mathrm{seconds} \end{array}$$

In addition to AUC, time to peak (i.e. the time from start of contrast injection until maximum signal), SI/s (i.e. the increase of signal intensity per second until maximum signal) and SI% (i.e. the increase in signal between start and peak, expressed in percent) was calculated for each ROI.

### Statistical methods and data management

Statistical comparisons in order to test differences between the symptomatic side and the contralateral side and between DEMRI 1 and DEMRI 2 was made by use of paired Student’s *t*-test for correlated variables. The Spearman rank order correlation was used in order to test independence between variables. The study employs multiple hypothesis testing, where each hypothesis was analyzed separately and the existence of patterns in and the consistency of the results were considered in the analysis. Probability values (p-value) <0.05 were considered significant.

## Results and discussion

### Comparison between symptomatic and contralateral ankle in the 14 patients with unilateral symptoms

In the fat ventrally of the symptomatic tendon, AUC, SI/s and SI% was significantly higher than that of the asymptomatic side before, but not after, eccentric training (Table 2). In the tendons there was no statistically significant difference of mean AUC, SI/s, SI% or time to peak between the symptomatic and the contralateral asymptomatic tendons before or after eccentric training. After, but not before training, there was a significantly shorter mean time to peak in the vessel on the symptomatic tendon side compared to the asymptomatic side (238 seconds (SD 97) and 289 seconds (SD 59) respectively, p < 0.05).

**Table 2:** Dynamic contrast enhancement in tendon and in fat ventrally of tendon

	**Symptomatic** **Before training**	**Contralateral** **Before training**	**Symptomatic** **After training**	**Contralateral** **After training**
AUC tendon	80 (65) NS	66 (59)	76 (53) NS	52 (65)
SI/s 10^3^ tendon	1.8 (1.5) NS	1.6 (0.8)	1.9 (1.2) NS	3.0 (4.7)
SI% tendon	41 (21) NS	38 (18)	38 (19) NS	33 (26)
AUC fat	31 (15)***	20 (11)	26 (15) NS	22 (13)
SI/s x 10^3^ fat	0.44 (0.24)*	0.32 (0.13)	0.43 (0.20) NS	0.34 (0.16)
SI% fat	14.5 (7.4)**	9.5 (4.9)	12.1 (6.3) NS	10.6 (5.4)

Mean AUC, SI/s and SI% in the symptomatic and contralateral side measured in the tendon and in the fat ventrally of the tendon before and after eccentric training, in 14 patients with unilateral symptoms. One standard deviation is provided within parenthesis. The significance of mean difference between the symptomatic and contralateral side has been noted. *** = p < 0.001, ** = p < 0.01, * = p < 0.05, NS = Not Significant.

### Comparison between before and after eccentric training in 20 patients

Mean AUC, SI/s, and SI% in tendon, fat and vessel did not change during eccentric training. Neither was there any significant change in time to peak in the symptomatic tendons. In the tendon of the contralateral side (the asymptomatic or the least symptomatic tendon) there was a significantly shorter time to peak after training (Table 3).

**Table 3:** Mean time to peak contrast enhancement

	**Symptomatic** **Before training**	**Contralateral** **Before training**	**Symptomatic** **After training**	**Contralateral** **After training**
Tendon	240 (89)	250 (78)	240 (100) NS	190 (110)*
Vessel	260 (76)	270 (61)	240 (89) NS	270 (78) NS
Fat	300 (64)	300 (57)	290 (53) NS	300 (42) NS

Mean time in seconds from start of contrast injection to peak contrast enhancement in tendon, vessel and in the fat ventrally of the tendon in the symptomatic and contralateral ankle of 20 patients with chronic achilles tendinosis before and after three months of eccentric calf-muscle training. One standard deviation is provided within parenthesis. The p-value comparing time from start to peak contrast enhancement before and after three months of eccentric training has been noted. * = p < 0.05, NS = Not Significant.

### Pain and performance

The median level of pain decreased from 5 to 3 after training (p < 0.01 in all 20 patients and p < 0.05 in 14 patients with unilateral symptoms). The median level of performance improved from 4 to 3 after training (p < 0.01 in all 20 patients and p < 0.001 in 14 patients with unilateral symptoms).

### Correlation to pain and performance

#### Tendon

AUC, time to peak, SI/s, or SI% in the symptomatic tendons (n = 20) did not correlate to the level of performance or pain neither before nor after the exercise programme. In the 14 contralateral non-symptomatic tendons, AUC and time to peak correlated to pain before training (r = 0.53 and r = 0.59 respectively, p < 0.05 both) but not after. SI/s or SI% in the 14 contralateral non-symptomatic tendons had no correlation to pain or performance.

#### Fat

In the 20 symptomatic sides, time to peak in the fat ventrally of the tendon correlated both to pain (r = 0.61, p < 0.01) and performance (r = 0.50, p < 0.05) after, but not before, training. Time to peak in the fat had an inverted correlation to performance after training (r = −0.68, p < 0.01) in the 14 contralateral non-symptomatic sides.

#### Vessel

In the 20 symptomatic sides SI/s in vessel correlated to performance before (r = 0.4, p < 0.05) but not after training. Time to peak had an inverted correlation to performance in the 20 symptomatic sides (r = −0.52, p < 0.05). In the 14 contralateral non-symptomatic sides AUC and SI/s in vessel had an inverted correlation to pain after training (r = −0.53 and r = −0.57 respectively, p < 0.05 both).

## Discussion

In this study dynamic contrast-enhanced MR imaging was performed in 20 patients with Achilles tendinosis before and after three months of eccentric calf-muscle training. In the fat ventrally of the tendon there was a significantly increased contrast enhancement in the symptomatic side compared to the contralateral non-symptomatic side before treatment but not after three months of treatment. However, in contradiction to this result there was no difference when comparing before to after treatment. Inside the tendon there was no difference of contrast enhancement in the symptomatic tendon compared to the contralateral tendon before or after the training programme. This finding is in disagreement with a previous study by Shalabi et al [23], and a study by Richards et al [20], where both these studies could show an increased AUC on the symptomatic tendon before treatment. This difference might be due to differences in duration of symptoms. The patients in Shalabi’s study had a median symptom duration of 12 months compared to the 31 month median duration in our study. In Richards’ study the duration was not reported. It might therefore be that the AUC had already returned to baseline due to the relatively long duration of the symptoms in our chronic tendinosis patients at the time of inclusion in our study.

In the present study a stronger contrast enhancement in the fat ventrally of the tendon in the symptomatic compared to the contralateral non-symptomatic tendon side was observed before, but not after three months of treatment. The observed contrast enhancement in the fat ventrally of the symptomatic tendon in the present study may be due to an increased vascularity but also to increased permeability because mean time to peak signal in the fat ventrally of the tendon in the symptomatic side was 40 seconds longer compared to the corresponding vessel (Table 3). Thus, it might be that the vascularity of the areas ventrally to the tendon [4], is an early marker of treatment effect, while the changes in the tendon need longer time to disappear. However, when evaluating the treatment effect by comparing the enhancement before with that of after training no significant effect could be observed, probably due to lack of statistical power. The clinical value of evaluating the vascularity of pretendinous fat is therefore low. The histopathological changes in Achilles tendinosis such as vascular proliferation, focal variation in cellularity, rounded nuclei, abnormal fibre structure, and increased areas of non-collagen extra cellular matrix have previously been shown to correlate with increased intratendinous signal at MRI of the tendon [2,11,17,18,10]. An increased contrast enhancement at MRI in pathologic tissue is thought to reflect an increased vascularity and/or an increased capillary permeability [16,17]. By using ultrasound and colour Doppler an increased vascularity mainly inside but also outside ventrally of the symptomatic tendon has previously been shown [4]. The observed contrast enhancement in the fat ventrally of the symptomatic tendon observed in the present study may thus be due to an increased vascularity. However, in the study by Shalabi et al [23] no correlation between histological increase in vascularity and increased AUC of contrast enhancement could be shown.

In a recent review of loading treatment programmes the majority of studies did not find any association between improved imaging parameters and clinical outcome in Achilles tendinosis [24]. A pilot study evaluating longitudinal microvascularisation of Achilles tendinosis showed that pain is not invariably associated with microvascularity [20]. This may explain the lack of correlation with symptoms in our study. Despite of a significant clinical improvement with both improved performance and decreased pain during the training programme there were almost no significant correlations between dynamic contrast enhancement in the symptomatic tendons to pain or performance. AUC and time to peak measured in the fat ventrally of the tendon at three months after treatment were related to pain and there was a relationship between AUC and SI/s measured in vessel before treatment to performance. There was no significant change of contrast enhancement in tendon, fat or vessel before compared to after 3 months of eccentric training.

In this study 20 patients with chronic achilles tendinosis were studied. In nine of those the tendinosis was sports related, while 11 were idiopathic. Although most of the sports related were due to running (n = 7) the heterogenous material in our study is a limitation. When our study was planned and conducted the questionnaire by Curwin and Stanish was used at our institution. That questionnaire is easier to use for the patients, but to enable comparisons with other studies it might have been better if the VISA-A questionnaire had been used.

The lack of correlation with symptoms indicates that dynamic enhanced MRI is currently not a useful method to evaluate chronic Achilles tendinosis compared to MRI without contrast media. Further development of imaging methods to visualize the pathology at chronic Achilles tendinosis is necessary. For example, the use of ultra short echo time (UTE) at MRI makes it possible to demonstrate features that are not apparent with conventional MRI sequences [25] which may make it possible to quantify the subtle signal changes at Achilles tendinosis and its treatment.

## Conclusion

In conclusion there was an increased contrast-media enhancement in the fat ventrally of the tendon in Achilles tendinosis that disappeared after three months of training, but otherwise dynamic contrast enhanced MRI did not show any additional value.

## Competing interests

The authors declare that they have no competing interests.

## Authors’ contributions

Study design was made by AG, AS and TM. Data acquisition was made by AG. Data analysis was made by AG and TB. The manuscript was drafted by AG and all authors have revised it critically. All authors read and approved the final manuscript.

## Pre-publication history

The pre-publication history for this paper can be accessed here:

http://www.biomedcentral.com/1471-2342/13/39/prepub
